# External validation and comparison of two delirium prediction models in patients admitted to the cardiac intensive care unit

**DOI:** 10.3389/fcvm.2022.947149

**Published:** 2022-08-03

**Authors:** Sung Eun Kim, Ryoung-Eun Ko, Soo Jin Na, Chi Ryang Chung, Ki Hong Choi, Darae Kim, Taek Kyu Park, Joo Myung Lee, Young Bin Song, Jin-Oh Choi, Joo-Yong Hahn, Seung-Hyuk Choi, Hyeon-Cheol Gwon, Jeong Hoon Yang

**Affiliations:** ^1^Division of Cardiology, Department of Medicine, Samsung Medical Center, Heart Vascular Stroke Institute, Sungkyunkwan University School of Medicine, Seoul, South Korea; ^2^Department of Critical Care Medicine, Samsung Medical Center, Sungkyunkwan University School of Medicine, Seoul, South Korea

**Keywords:** delirium prediction, prediction model, cardiac intensive care unit, Early PREdiction of DELIRium, PREdiction of DELIRium

## Abstract

**Background:**

No data is available on delirium prediction models in the cardiac intensive care unit (CICU), although preexisting delirium prediction models [PREdiction of DELIRium in ICu patients (PRE-DELIRIC) and Early PREdiction of DELIRium in ICu patients (E-PRE-DELIRIC)] were developed and validated based on a population admitted to the general intensive care unit (ICU). Therefore, we externally validated the usefulness of the PRE-DELIRIC and E-PRE-DELIRIC models and compared their predictive performance in patients admitted to the CICU.

**Methods:**

A total of 2,724 patients admitted to the CICU were enrolled between September 2012 and December 2018. Delirium was defined as at least one positive Confusion Assessment Method for the ICU (CAM-ICU) which was screened at least once every 8 h. The PRE-DELIRIC value was calculated within 24 h of CICU admission, and the E-PRE-DELIRIC value was calculated at CICU admission. The predictive performance of the models was evaluated by using the area under the receiver operating characteristic (AUROC) curve, and the calibration slope was assessed graphically by plotting.

**Results:**

Delirium occurred in 677 patients (24.8%) when the patients were assessed thrice daily until 7 days of the CICU stay. The AUROC curve for the prediction of delirium was significantly greater for PRE-DELIRIC values [0.84, 95% confidence interval (CI): 0.82–0.86] than for E-PRE-DELIRIC values (0.79, 95% CI: 0.77–0.80) [z score of −6.24 (*p* < 0.001)]. Net reclassification improvement for the prediction of delirium increased by 0.27 (95% CI: 0.21–0.32, *p* < 0.001). Calibration was acceptable in the PRE-DELIRIC model (Hosmer-Lemeshow *p* = 0.170) but not in the E-PRE-DELIRIC model (Hosmer-Lemeshow *p* < 0.001).

**Conclusion:**

Although both models have good predictive performance for the development of delirium, even in critically ill cardiac patients, the performance of the PRE-DELIRIC model might be superior to that of the E-PRE-DELIRIC model. Further studies are required to confirm our results and design a specific delirium prediction model for CICU patients.

## Introduction

Delirium, defined as acute brain dysfunction characterized by a fluctuating disturbance of attention, awareness, and cognitive function caused by an underlying medical condition, is associated with prolonged hospital admission, increased health care costs, and substantially increased morbidity and mortality (1, 2). Therefore, efforts have been made to detect delirium in patients who may benefit from delirium prevention, and several delirium prediction models have been developed in patients admitted to a medical and/or mixed intensive care unit (ICU).

Based on the data from the general ICU patients, the PREdiction of DELIRium in ICu patients (PRE-DELIRIC) model was developed using 10 predictors that could be obtained within 24 h of ICU admission (3). For the early prediction of the risk of delirium in ICU patients, the Early PREdiction of DELIRium in ICu patients (E-PRE-DELIRIC) model was developed using nine predictors that could be collected at the time of ICU admission (4). Both models included data on age, blood urea concentrations, need for urgent admission, and main diagnosis at the time of admission as predictors. The PRE-DELIRIC model included data on the severity score, the use of opioids and sedatives, metabolic acidosis, mentality, and infection, and the E-PRE-DELIRIC model included data on the history of cognitive impairment and alcohol abuse, mean arterial blood pressure, use of corticosteroids, and present of respiratory failure, as additional predictors. These models showed acceptable performance in different cohorts and patients admitted to the general ICU (5–7).

Cardiac ICUs (CICUs) have evolved from units that focus exclusively on patients with acute myocardial infarction to units that provide comprehensive critical care for patients with various cardiovascular diseases, patients requiring hemodynamic monitoring, and patients with mechanical circulatory devices for cardiogenic shock (8). Furthermore, age, the severity of illness, and comorbidities in patients admitted to the CICU have increased, and patients requiring admission to the ICU and CICU overlap significantly (9). Accordingly, delirium, which has been reported to have prognostic implications, is a common symptom in critically ill cardiac patients (10). However, to date, there has been no evidence that delirium prediction models validated in the general ICU apply to patients admitted to the CICU.

Therefore, we sought to investigate the usefulness of the PRE-DELIRIC and E-PRE-DELIRIC models for detecting the risk of developing delirium and compare the predictive performance of the two models in patients admitted to the CICU.

## Methods

### Study population and cardiac intensive care unit setting

**Figure 1:**
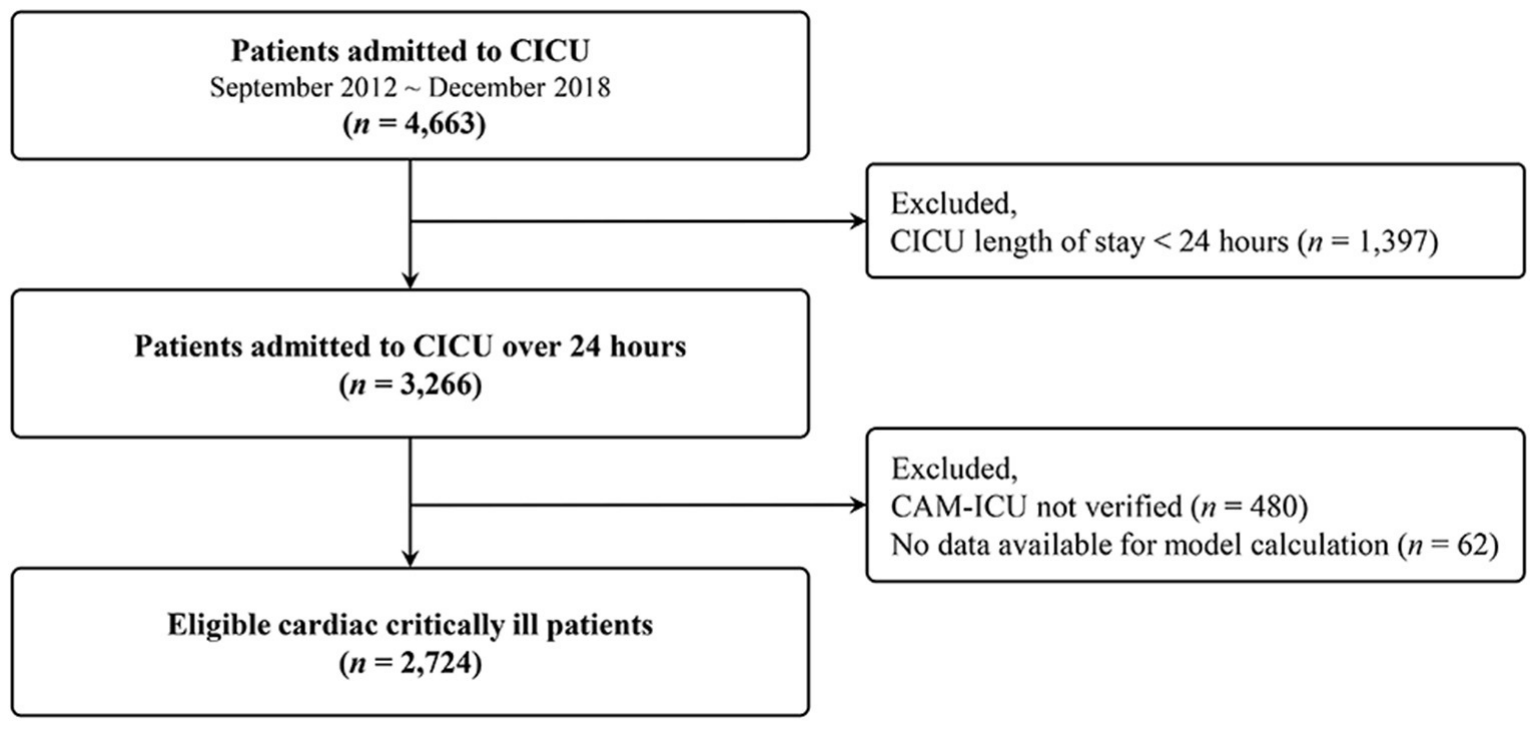
Study flow diagram. CICU, cardiac intensive care unit; CAM-ICU, confusion assessment method for the intensive care unit; LOS, length of stay.

A retrospective cohort study was conducted and this study included 4,663 patients aged >18 years who were admitted to the CICU between September 2012 and December 2018. Exclusion criteria were as follows; (1) Patients who stayed in the ICU for <24 h (*n* = 1,397), (2) those who could not complete delirium assessment (*n* = 480), or (3) those whose data for calculation of delirium prediction models were not available (*n* = 62). Finally, 2,724 patients were included in this analysis (Figure 1).

Our CICU is a 12-bed unit that provides comprehensive critical care to patients with various cardiovascular diseases. Details of the Samsung Medical Center's CICU setting have been described in previous reports (11, 12). In brief, the CICU provides non-invasive and invasive devices for monitoring the hemodynamic and cardiovascular support statuses, including mechanical ventilation and extracorporeal membrane oxygenation (ECMO). The nurse-to-patient ratio is 1:2, and level 1 care is provided, which is managed by a dedicated cardiac intensivist (13). Cardiac surgery support is readily available. In addition, multidisciplinary care is provided through consultation with a dietitian, pharmacist, and respiratory care practitioner. For general intensive care, clinical practice guidelines published by the Society of Critical Care Medicine are adopted (14).

This study was approved by the Institutional Review Board of Samsung Medical Center. The requirement for written informed consent was waived because of the retrospective nature of the study (IRB No. 2020-10-102).

### Data collection and definitions

Clinical, laboratory, and outcome data were collected by a trained study coordinator after reviewing the medical records. The confusion assessment method for the ICU (CAM-ICU) was performed by nurses thrice a day in patients with a Richmond agitation-sedation scale score of −3 (indicating movement or eye-opening to voice but no eye contact) or higher (15). Delirium was defined according to the CAM-ICU. The CAM-ICU was screened at least once every 8 h by well-trained intensive care nurses, and the recorded CAM-ICU value was re-checked daily by a senior nurse. In this study, we evaluated delirium within 7 days of the CICU stay. The PRE-DELIRIC model was calculated using predictors within 24 h of CICU admission, and the E-PRE-DELIRIC model was evaluated using predictors at CICU admission (3, 4). The detail of the calculation of PRE-DELIRIC and E-PRE-DELIRIC model was described in Supplementary Table 1.

### Statistical analysis

The data are summarized using descriptive statistics: numbers and percentages are calculated for categorical variables, while continuous variables are summarized as medians with interquartile ranges (IQR; 25^th^ and 75^th^ percentiles). The discriminative power of the two models was assessed using the area under the receiver operating characteristic curve (AUROC), and their predictive performance was compared using DeLong's test (16). Calibration was assessed graphically by plotting the observed outcome frequencies against the mean predicted outcome probabilities or risks. For all analyses, statistical significance was set at *p* < 0.05. Statistical analyses were performed using SAS version 9.4 (SAS Institute Inc., Cary, NC, USA) and R 4.0.2 (Vienna, Austria; http://www.R-project.org).

## Results

### Patient characteristics

Table 1 provides an overview of the characteristics of the CICU patients. The median age was 67.0 (IQR 56.0–76.0) years, and 64.6% of the patients were males. The most common underlying disease was hypertension (55.5%), followed by diabetes mellitus (33.0%) and solid cancer (12.4%). Stroke (11.0%) was the most common underlying neurological disease, and 2.8% of all patients had a history of cerebral hemorrhage. Patients were commonly admitted from the emergency room (59.0%) and admitted for medical management (98.0%). Acute coronary syndrome (43.8%), heart failure (30.6%), and arrhythmia (11.2%) were the most frequent diagnoses at CICU admission. The median Sequential Organ Failure Assessment score was 3.0 (IQR 2.0–6.0). Two hundred and seventy-two (9.9%) patients received cardiopulmonary resuscitation at CICU admission, and vasoactive drugs were used in 16.3%, mechanical ventilators in 13.8%, and ECMO in 4.6% of the patients.

**Table 1:** Clinical characteristics of the study population.

**Variables**	**CICU patients (*n* = 2,724)**
Age, years	67.0 (56.0–76.0)
Sex, male	1,760 (64.6)
Body mass index, kg/m^2^	23.8 (21.8–25.7)
Underlying disease	
Hypertension	1,516 (55.6)
Diabetes	892 (33.0)
Chronic lung disease	176 (6.5)
End stage renal disease on hemodialysis	114 (4.2)
Liver disease	72 (2.6)
Solid cancer	338 (12.4)
Hematologic malignancy	10 (0.4)
Stroke	296 (11.0)
Cerebral hemorrhage	76 (2.8)
Dementia	41 (1.5)
Psychotic disorder	5 (0.2)
Brain tumor	6 (0.2)
Chronic neurodegenerative disease	46 (1.7)
Admission route	
Emergency room	1,604 (59.0)
General ward	399 (14.5)
High dependency unit	4 (0.1)
Other intensive care unit	283 (10.2)
Outpatient clinic	183 (6.7)
Other hospital	251 (9.2)
Admission cause	
Medical	2,671 (98.0)
Surgical	53 (2.0)
Primary diagnosis	
Acute coronary syndrome	1,192 (43.8)
Heart failure	833 (30.6)
Arrhythmia	305 (11.2)
Aortic disease	170 (6.2)
Pulmonary hypertension	121 (4.4)
Pericardial disease	75 (2.8)
Other	28 (1.0)
Initial SOFA	3.0 (2.0–6.0)
Cardiac arrest at admission	272 (9.9)
Organ support at admission	
Vasoactive drug	444 (16.3)
Mechanical ventilation support	377 (13.8)
Intra-aortic balloon pump	29 (1.1)
Extracorporeal membrane oxygenation	125 (4.6)
Delirium occurrence in CICU	
Outcomes	677 (24.8)
CICU length of stay	2.1 (1.6–4.0)
CICU mortality	115 (4.2)
Hospital mortality	184 (6.8)

Presented values are medians with interquartile ranges or numbers with percentages.

CICU, cardiac intensive care unit; SOFA, sequential organ failure assessment.

CICU patient characteristics according to the variables of the delirium prediction models are shown in Table 2. Of the 2,724 patients, 15.1% had drug-induced coma, and 8.8% were in coma due to miscellaneous within 24 h of CICU admission. In addition, 506 (20%) patients used opioids, and 1,315 (48.2%) patients received sedative drugs within 24 h of CICU admission. These data were used to calculate the PRE-DELIRIC model values. At CICU admission, 1,412 (52.0%) patients presented with respiratory failure, and 38 (1.0%) patients used corticosteroids. These data were also used to calculate the E-PRE-DELIRIC model values.

**Table 2:** Patient characteristics of the study with delirium assessment tools according to the original delirium study.

**Variables**	**CICU patients (*n* = 2,724)**
**PRE-DELIRIC**	
Age, years	67.0 (56.0–76.0)
Blood urea nitrogen, mg/dL	21.1 (15.0–33.3)
ICU admission category	
Medical	2671 (98.0)
Surgical	53 (2.0)
Urgent admission	383 (14.1)
Coma types	
None	2,072 (76.1)
Drug-induced	411 (15.1)
Miscellaneous	241 (8.8)
Infection	35 (1.3)
Cumulative morphine use	
0.01–7.1 mg/day	289 (11.0)
7.2–18.6 mg/day	163 (6.0)
≥18.7 mg/day	54 (2.0)
Sedative use	1,315 (48.2)
APACHE II score	10.0 (6.0–15.0)
Metabolic acidosis	210 (7.7)
**E-PRE-DELIRIC**	
Age, years	67.0 (56.0–76.0)
Blood urea nitrogen, mg/dL	21.1 (15.0–33.3)
Urgent admission	383 (14.1)
Alcohol abuse	157 (5.7)
Cognitive impairment	421 (15.1)
ICU admission category	
Medical	2671 (98.0)
Surgical	53 (2.0)
Mean blood pressure, mmHg	89.0 (76.0–103.0)
Corticosteroid use	38 (1.0)
Respiratory failure	1,412 (52.0)

Presented values are medians with interquartile ranges or numbers with percentages.

CICU, cardiac intensive care unit; PRE-DELIRIC, PREdiction of DELIRium in ICu patients; ICU, intensive care unit; APACHE, acute physiology and chronic health evaluation; E-PRE-DELIRIC, Early PREdiction of DELIRium in ICu patients.

### Predictive performances of the PRE-DELIRIC and E-PRE-DELIRIC models

**Figure 2:**
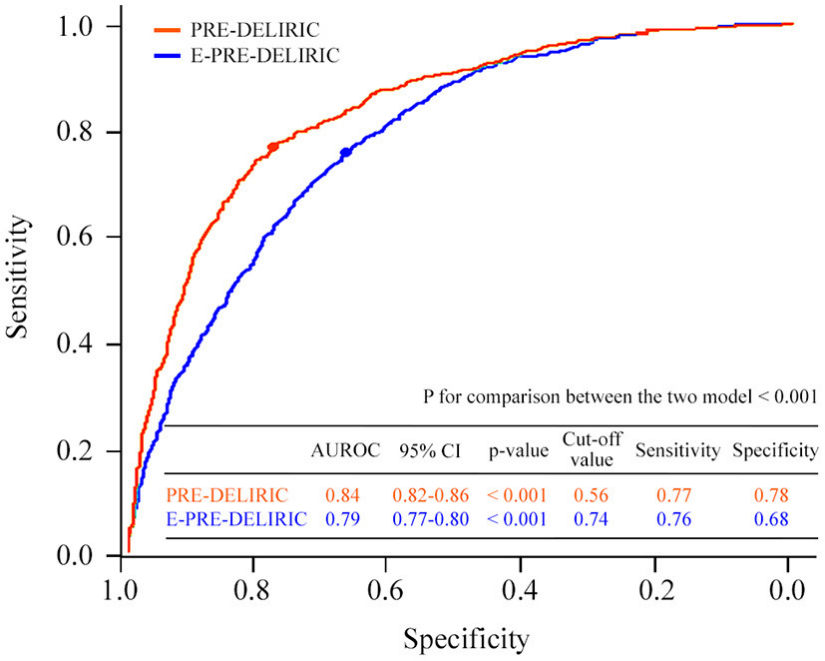
Comparison of the predictive performances of the two delirium models. AUROC, area under the receiver operating characteristic; PRE-DELIRIC, PREdiction of DELIRium in ICu patients; E-PRE-DELIRIC, Early PREdiction of DELIRium in ICu patients.

**Figure 3:**
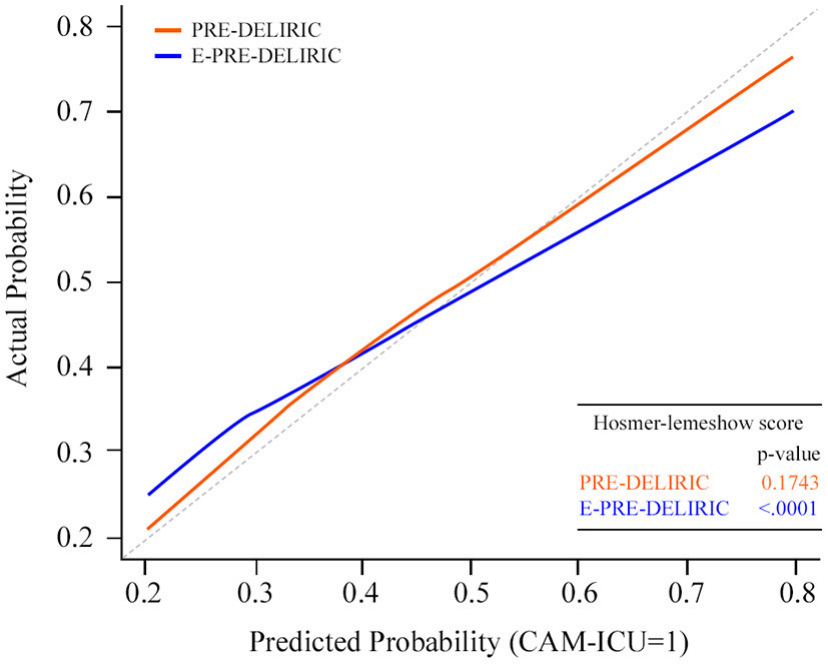
Calibration plot of both prediction models. Calibration plot [with 95% confidence interval (CI), blue line] by 10 prediction values for the PRE-DELIRIC model. Calibration plot (with 95% CI, red line), using nine prediction values for the E-PRE-DELIRIC model. The dashed line indicates perfect calibration. PRE-DELIRIC, PREdiction of DELIRium in ICu patients; E-PRE-DELIRIC, Early PREdiction of DELIRium in ICu patients.

Of all the patients, 677 (24.8%) experienced delirium during CICU admission within 7 days. Overall, 155 (4.2%) patients died in the CICU, and the hospital mortality rate was 6.8%. The predictive performance for the development of delirium and its comparison for the two models are presented in Figure 2. The AUROC of the PRE-DELIRIC model was 0.84 [95% confidence interval (CI): 0.82–0.86, *p* < 0.001], and the cut-off point was 0.56 (sensitivity and specificity were 0.68 and 0.78, respectively). The AUROC of the E-PRE-DELIRIC model was 0.79 (95% CI: 0.77–0.80, *p* < 0.001), and the cut-off point was 0.74 (sensitivity and specificity were 0.76 and 0.77, respectively). The PRE-DELIRIC model showed a significantly better statistical performance than the E-PRE-DELIRIC model for predicting the development of delirium [z score of −6.25 (*p* < 0.001)]. The PRE-DELIRIC and E-PRE-DELIRIC models were well-calibrated; however, calibration was only acceptable in the PRE-DELIRIC model (Hosmer-Lemeshow *p* = 0.17) but not in the E-PRE-DELIRIC model (*p* < 0.001) (Figure 3).

## Discussion

We externally validated the usefulness of two delirium prediction models (PRE-DELIRIC and E-PRE-DELIRIC) and compared their performance in CICU patients. The major findings of this study were that both prediction models for the development of delirium had fair to good predictive performance even in critically ill cardiac patients; in particular, the PRE-DELIRIC model predicted the development of delirium better and was well-calibrated. To the best of our knowledge, this is the first study to apply preexisting delirium prediction models to patients admitted to the CICU.

Some differences between the general ICU and CICU remain, such as the patient population, ICU environment, management of mechanical circulatory support devices, and medication prescriptions. However, since coronary care units were designed in the 1960's to monitor and treat the acute phase of myocardial infarction, profiles of patients with cardiovascular disease admitted to these units are gaining complexity and diversity (17). In addition, patients admitted to the CICU are often elderly and present with multiple comorbidities. Therefore, the incidence of delirium in the CICU has substantially increased (18), and several studies have shown that delirium is associated with morbidity and mortality in patients admitted to the CICU (19, 20). Falsini et al. reported that delirium is associated with a long and complicated hospital stay and increased mortality in elderly CICU patients (19). Similar results were found in a nationwide data analysis of patients with myocardial infarction in the United States (20). Recently, studies on delirium in patients admitted to the CICU have been actively conducted; however, there has been no study that validated delirium prediction models in the general ICU for patients admitted to the CICU.

The PRE-DELIRIC model was developed and validated in 2,361 mixed ICU patients, and this model comprised 10 variables that were available within 24 h after ICU admission. The AUROC for temporal validation showed good performance (3). However, up to 25% of ICU patients develop delirium within 24 h of admission to the ICU; therefore, a model for the early detection of the risk of developing delirium was needed (21). The E-PRE-DELIRIC model was developed for this purpose at the time of ICU admission. The E-PRE-DELIRIC model was developed and validated in 2,914 patients at 13 ICUs from 7 countries and comprised nine variables that could be evaluated upon admission to the ICU. In this study, we evaluated the usefulness of the PRE-DELIRIC and E-PRE-DELIRIC models for detecting the risk of developing delirium; the PRE-DELIRIC model showed good performance, and the E-PRE-DELIRIC model showed fair performance in critically ill cardiac patients. One unanticipated finding was that the performance of the two models for patients admitted to the CICU was better than that for patients admitted to the general ICU, as reported in a previous study (5). In particular, the PRE-DELIRIC model showed better accuracy than the E-PRE-DELIRIC model documented using a calibration plot, and the results were similar to those of a previous study conducted in a general ICU (5). Since our study was conducted in a large-scale cohort admitted to the CICU in the era of mechanical circulatory support, these findings reflect the modern CICU environment, and the two delirium prediction models might be useful in CICU patients, as well as general or mixed ICU patients.

Delirium prevention involves understanding predisposing and precipitating risk factors. The development of delirium is complex and depends on several non-modifiable and modifiable clinical factors (22). Therefore, understanding the characteristics of patients admitted to the CICU is important. First, patients with acute heart failure complicated by coexisting acute and chronic comorbidities have increased in the CICU (23), and these patients are highly vulnerable and more elderly. In this study, we also showed that 30.6% of all CICU patients were admitted for the management of heart failure. Patients with low cardiac output due to pump failure might be easily affected by invasive procedures, sedatives, sleep deprivation, and medications during CICU stay. In addition, a previous study reported that heart failure alone was an independent risk factor for delirium (24). Second, various temporary and permanent mechanical supports and advanced procedures are often applied to critically ill cardiac patients (25, 26). These interventions might induce prolonged immobility and increased need for sedatives, resulting in an increased occurrence of delirium (27). Third, since patients with cardiogenic shock are easily affected by cerebral hypo-perfusion, which is related to systemic hypotension known to be associated with the development of delirium, delirium might be a common issue in modern and future CICUs with advanced medical devices (28, 29). Finally, the length of ICU stay differs between the CICU and medical ICU (30, 31). Indeed, there are certain differences, such as the patient characteristics, ICU environments, and ICU stay period between patients admitted to the CICU and medical or mixed ICU. The incidence rate of delirium according to age, CICU length of stay, and SOFA score were described as bar plots (Supplementary Figure 1). Accordingly, further work is required to develop a specific delirium prediction model for critically ill cardiac patients with good performance, given the unique characteristics of patients admitted to the CICU.

Our study has several limitations. First, it was subject to a selection bias, which may have influenced our findings due to its retrospective and single-center observational nature. Second, the development of delirium was counted until 7 days of ICU stay because it was relatively shorter in the CICU than in the general or mixed ICU. Only 323 (6.9%) patients were admitted for over 7 days in our cohort. Third, admission categories in our study were limited to only two types, medical and surgical, based on the CICU characteristics, while there are four types of admission categories for delirium predictive values, such as medical, surgical, traumatic, and neurological/neurosurgical in previous studies that developed two delirium models (3, 4). Forth, although delirium can be evaluated with various tools, the CAM-ICU was only used to evaluation in our CICU. Therefore, discriminative power of two models might be changed when delirium was assessed with other tools. Lastly, one of the variables for the PRE-DELIRIC model, cognitive impairment, was not evaluated by the cognitive impairment assessment tools.

## Conclusion

The PRE-DELIRIC model has an excellent predictive performance for the development of delirium and its performance is better than that of the E-PRE-DELIRIC model, although the E-PRE-DELIRIC model also has good predictive performance in critically ill cardiac patients admitted to the CICU. Further studies are required to confirm our results and design a specific delirium prediction model for CICU patients.

## Data availability statement

The original contributions presented in the study are included in the article/Supplementary material, further inquiries can be directed to the corresponding author.

## Ethics statement

This study was approved by the Institutional Review Board of Samsung Medical Center. The requirement for written informed consent was waived because of the retrospective nature of the study (IRB No. 2020-10 102).

## Author contributions

SK, R-EK, and JY: data collection, statistical analysis, manuscript draft, and responsible for the overall content as guarantors. All authors: data analysis and interpretation, critical revision, editing, and approval of the final manuscript.

## Conflict of interest

The authors declare that the research was conducted in the absence of any commercial or financial relationships that could be construed as a potential conflict of interest.

## Publisher's note

All claims expressed in this article are solely those of the authors and do not necessarily represent those of their affiliated organizations, or those of the publisher, the editors and the reviewers. Any product that may be evaluated in this article, or claim that may be made by its manufacturer, is not guaranteed or endorsed by the publisher.

## Abbreviations

AUROC: area under the receiver operating characteristic curve
CAM-ICU: confusion assessment method for the intensive care unit
CI: confidence interval
CICU: cardiac intensive care unit
ECMO: extracorporeal membrane oxygenation
E-PRE-DELIRIC: Early PREdiction of DELIRium in ICu patients
ICU: intensive care unit
IQR: interquartile ranges
PRE-DELIRIC: PREdiction of DELIRium in ICu patients.
