# Supplementary material for: External validation and comparison of two delirium prediction models in patients admitted to the cardiac intensive care unit
Source: Front Cardiovasc Med. 2022 Aug 3;9:947149. doi: 10.3389/fcvm.2022.947149 (PMC9382019; doi:10.3389/fcvm.2022.947149)

**Supplementary Appendix**

**External Validation and Comparison of Two Delirium Prediction Models in Patients Admitted to the Cardiac Intensive Care Unit**

Sung Eun Kim, MD^1^*, Ryoung-Eun Ko, MD^2^*, Jeong Hoon Yang, MD^1, 2^

**Table of Contents**

- **Supplementary Tables**
- **Supplementary Figure and Figure Legends**

**Supplemental Tables**

**Supplemental Table 1. Formula for PRE-DELIRIC MODEL and E-PRE-DELIRIC MODEL**

| **PRE-DELIRIC MODEL** |  |
| --- | --- |
| **Risk of delirium = 1/(1+exp-(-6.31)** |  |
| Age | 0.04 |
| APACHE-II score | 0.06 |
| COMA |  |
| None | 0 |
| Drug-induced | 0.59 |
| Miscellaneous | 2.92 |
| Combination | 3.06 |
| Admission category |  |
| Surgery | 0 |
| Medical | 0.31 |
| Infection | 1.05 |
| Metabolic acidosis | 0.29 |
| Morphine use |  |
| 0.01 - 7.1 mg/day | 0.41 |
| 7.2 – 18.6 mg/day | 0.13 |
| >18.6 mg/day | 0.51 |
| Sedation | 1.3 |
| Blood urea nitrogen, mg/dL | 0.03 |
| Urgent admission | 0.40 |
| **E-PRE-DELIRIC MODEL** |  |
| **Risk of delirium = 1/(1+exp-(-3.91)** |  |
| Age | 0.03 |
| Cognitive impairment | 0.88 |
| Alcohol abuse | 0.51 |
| Admission category |  |
| Surgery | 0 |
| Medical | 0.37 |
| Urgent admission | 0.61 |
| Mean arterial pressure | -0.01 |
| Corticosteroid use | 0.28 |
| Respiratory failure | 0.98 |
| Blood urea nitrogen, mg/dL | 0.02 |

APACHE = Acute physiology and chronic health evaluation

Ten variables for PRE-DELIRIC MODEL

Nine variables for E-PRE-DELIRIC MODEL

**Supplemental Figure Legends**

A. Incidence rate of delirium with age, B. Incidence rate of delirium with CICU length of stay, C. Incidence rate of delirium with SOFA score.

Abbreviations : CICU, cardiac intensive care unit; SOFA, sequential organ failure assessment

**Supplemental Figures**

**Supplemental Figure 1. Incidence rate of delirium**

**A B C**


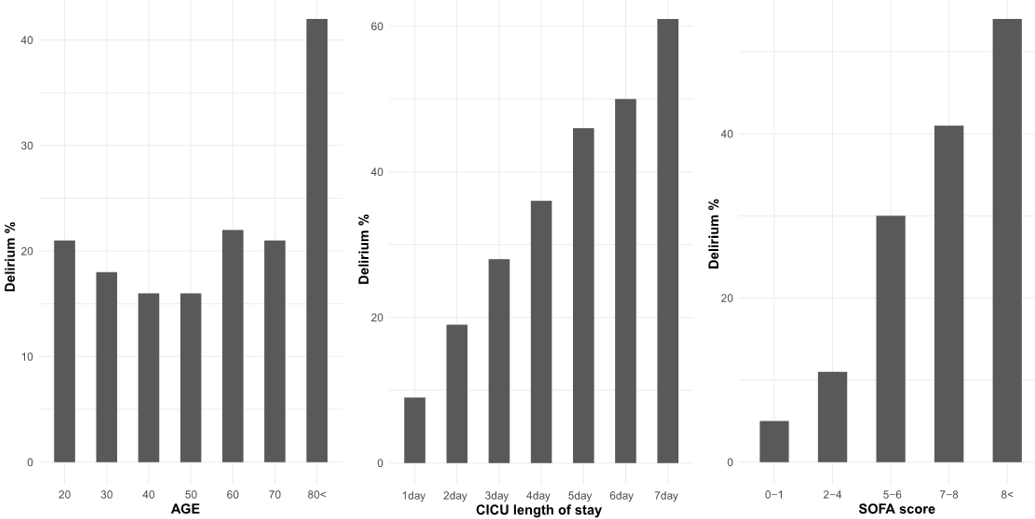

Supplement: Supplementary file 1 [file Data_Sheet_1.docx]
